# Effect size – large, medium, and small

**DOI:** 10.1007/s40037-016-0308-y

**Published:** 2016-10-17

**Authors:** Jimmie Leppink, Patricia O’Sullivan, Kal Winston

**Affiliations:** 1 University Maastricht, Maastricht, The Netherlands; 2 University of California, San Francisco, USA; 3 The Commonwealth Medical College, Pennsylvania, USA

## Abstract

The overall purpose of the ‘Statistical Points and Pitfalls’ series is to help readers and researchers alike increase awareness of how to use statistics and why/how we fall into inappropriate choices or interpretations. We hope to help readers understand common misconceptions and give clear guidance on how to avoid common pitfalls by offering simple tips to improve your reporting of quantitative research findings. Each entry discusses a commonly encountered inappropriate practice and alternatives from a pragmatic perspective with minimal mathematics involved. We encourage readers to share comments on or suggestions for this section on Twitter, using the hashtag: #mededstats.

In this entry, we examine a statistical measure commonly cited in educational research, effect sizes: what they are, when they are needed, and challenges with their interpretation. While effect sizes are encountered in many research articles in medical education, it is often not really clear why they are reported and what they mean in the context of the study. We argue that effect sizes are useful only if a research question calls for them. They should be presented along with the statistics they are calculated from, and should be reported with confidence intervals when generalizing study findings to a broader population.

## Example study

Consider a randomized controlled experiment that compares two learning formats – groups A (treatment) and B (control) – in terms of exam performance on a scale ranging from 0 to 80. Suppose, the average exam performance is 50 points in group A and 55 points in group B. In other words, the difference between means is 5 points (55–50). Suppose, the standard deviation of exam scores is about 10 points in both groups. Expressed in standard deviations, the group difference is 0.5: mean difference/standard deviation = 5/10. This indicates a ‘medium’ size difference: by convention, differences of 0.2, 0.5, and 0.8 standard deviations are considered ‘small’, ‘medium’, and ‘large’ *effect sizes *respectively [1].

## Different scales

In order to understand the idea of effect sizes, we need to understand the problem of different scales. In educational research, we are often concerned with differences obtained on different scales in questionnaires and assessments, such as exams with different numbers of questions in different empirical studies. In contrast to a variable such as temperature, where a difference of 18 °F corresponds with a difference of 10 °C, the aforementioned 5 points of difference in exam score on a scale from 0 to 80 does not necessarily correspond with 10 points of difference in exam score on a scale that ranges from 0 to 160. Different exams may have questions of different difficulty, while a longer exam may include additional aspects of difficulty, such as stamina and speed.

## What are effect sizes?

Despite the challenges with equating differences obtained on different scales, researchers are sometimes interested in comparing differences obtained in different studies (e. g. meta-analysis [1]), with different participants and different scales. Moreover, sometimes researchers are interested in comparing the impact of different treatments on a response variable of interest, such as the aforementioned exam performance. For these purposes, researchers can calculate so-called *effect sizes*, that is: differences of interest expressed in statistical units such as standard deviations [1].

## Effect sizes, like other statistics, fluctuate from study to study

Different empirical studies include different participants. Therefore, means and standard deviations – like all statistics – fluctuate from study to study [2–4]. Since effect sizes are calculated from study statistics, such as the difference of 0.5 standard deviations between group A and group B observed in our experiment, effect sizes fluctuate from study to study as well [4]. Thus, whether we report a difference in exam score on a scale from 0 to 80 (5 points with a standard deviation of 10) or in standard deviations (0.5), we should be aware of the uncertainty around these estimates due to these fluctuations, and report *confidence intervals* around them whenever we intend to generalize study findings to the population we sampled our participants from [4].

## Confidence intervals around effect sizes

The extent to which a statistic, such as a difference between means, fluctuates from study to study is expressed by the *standard error* [2]. This standard error plays an important role in statistical tests and in confidence intervals. A 95 % confidence interval around a statistic, such as a difference between means, extends to about twice the standard error (i. e. *margin of error*) to either side of the statistic [2]. If, for instance, the margin of error is 7, the 95 % confidence interval for the difference between means extends from −2 to 12 (i. e. 5+/−7). Given that a difference between means of 5 points is a difference of 0.5 standard deviations, the 95 % confidence interval for the difference expressed in standard deviations (i. e. effect size) extends from −0.2 to 1.2 (i. e. dividing −2 and 12 by 10, respectively). The number of −0.2 indicates a ‘small’ size difference in one direction, whereas the number of 1.2 indicates a ‘large’ size difference in the other direction [1].

## Confidence intervals that include the value ‘0’

The example just provided indicates that if ‘0’ lies in the confidence interval for the difference between means on a scale from 0 to 80, the confidence interval for the difference between means expressed in standard deviations will also include the value ‘0’. In either case, the confidence interval indicates that, in a replication of the study, the difference between means – whether expressed on a scale from 0 to 80 or in standard deviations – could be very different. In other words, labelling differences in terms of effect size does not resolve the problem of small samples [2].

## Meaningful interpretation of an effect size

Researchers in medical education frequently conduct studies with particular theory and previous research in mind. For instance, a particular hypothesis about a treatment effect (i. e. the difference between means of groups A and B) typically forms the starting point for the example experiment. Based on previous research, researchers may even have specific expectations with regard to that treatment effect, such as about 1 standard deviation difference between the two groups. In this context, reporting the effect size of 0.5 standard deviations (mean difference/standard deviation = 5/10) is meaningful. However, the researchers should still express the difference of interest on the scale from 0 to 80 as well, for the latter can facilitate our understanding of the meaning of that difference in the context in which it is used. For instance, had the means of the two groups been 50 and 51, with a standard deviation of about 2 in each group, that would also have resulted in an effect size of 0.5 standard deviations. However, a difference between 50 and 51 may or may not have useful implications for educational practice or further research. Thus, we recommend to carefully consider if a particular research question calls for an effect size (e. g. half a standard deviation) and, if so, to report the actual difference (e. g. 5 points with a standard deviation of 10) as well. Effect sizes do not mean a lot when the actual difference is too small for practical purposes.

In some cases, a study includes multiple treatment groups. For instance, researchers are interested in the effects of two treatments and therefore conduct an experiment with two treatment groups and a control group, hence three groups in total. In such a study, researchers may have hypotheses about the difference between each pair of groups (i. e. three pairs in total). In this context, effect sizes that focus on comparisons of two groups – here: how many standard deviations difference between each pair of groups – are more meaningful than effect sizes calculated for all differences across all groups together [4].

## To conclude

Effect sizes only have added value over and above actual differences if a particular research question is stated in terms of effect sizes and even then they are preferably interpreted along with the actual differences. Calculated from actual differences, effect sizes are subject to the same study-to-study fluctuation, and should thus be reported with confidence intervals when generalizing study findings to a broader population.

## Note

For effect size calculations, it is important to consider a number of aspects: the type of variables involved (e. g. categorical or scale), the type of statistical method used (e. g. Chi-square, correlation, regression), eventual departures from starting assumptions (e. g. groups having very different standard deviations), and to some extent sample size [1, 4]. It is recommended to consult a good book [1], article [5] or online effect size calculator [6] that allows researchers to take these aspects into account.
